# Long Term Metabolic Effects of Sacubitril/Valsartan in Non-Diabetic and Diabetic Patients With Heart Failure Reduced Ejection Fraction: A Real Life Study

**DOI:** 10.3389/fphys.2022.897109

**Published:** 2022-05-25

**Authors:** Giuseppe Armentaro, Graziella D’Arrigo, Sofia Miceli, Velia Cassano, Maria Perticone, Raffaele Maio, Alberto Maria Marra, Franco Arturi, Antonio Cittadini, Giovanni Tripepi, Giorgio Sesti, Angela Sciacqua

**Affiliations:** ^1^ Department of Medical and Surgical Sciences, University Magna Græcia of Catanzaro, Catanzaro, Italy; ^2^ CNR-IFC, Istituto di Fisiologia Clinica, Clinical Epidemiology and Physiopathology of Renal Diseases and Hypertension, Reggio Calabria, Italy; ^3^ Department of Translational Medical Sciences, “Federico II” University Hospital and School of Medicine, Naples, Italy; ^4^ Department of Clinical and Molecular Medicine, University Rome-Sapienza, Naples, Italy; ^5^ Research Center for the Prevention and Treatment of Metabolic Diseases, University of Catanzaro, Catanzaro, Italy

**Keywords:** sacubitril/valsartan, type 2 diabetes mellitus, HbA1c, cardiac index, global longitudinal strain, heart failure with reduced ejection fraction

## Abstract

Sacubitril/Valsartan (sac/val) has improved clinical prognosis in patients affected by heart failure (HF) with reduced ejection fraction (HFrEF). HF and type 2 diabetes mellitus (T2DM) frequently coexist, with a prevalence of T2DM of 35%–40% in patients with HF. T2DM is the third co-morbidities in patients with HF and a strong independent risk factor for the progression of HF. In a post hoc analysis of PARADIGM-HF, improved glycemic control was shown in patients with T2DM and HFrEF receiving sac/val compared to enalapril at 12 months of follow-up. The aim of the present study was to evaluate, in a series of repeated observations in 90 HFrEF patients, the long term effect of sac/val treatment on renal function, glycometabolic state and insulin sensitivity parameters, according to diabetic status. We studied 90 patients (74 men and 16 women, mean age 68 ± 10 years, 60 diabetics and 30 non-diabetics) suffering from HFrEF and still symptomatic despite optimal pharmacological therapy. Patients with left ventricular ejection fraction (LVEF) <35% and II-III NYHA functional class were enrolled. All patients underwent clinical-instrumental and laboratory determinations and Minnesota Living with HF Questionnaire (MLHFQ) every 6 months until 30 months to evaluate benefits and adverse events. After 30 months follow-up, we observed a significant improvement in glycometabolic parameters including HbA1c, fasting glucose and insulin, insulin-like growth factor-1 (IGF-1), HOMA index, and LDL cholesterol. Moreover, renal function, NTpro-BNP levels and echocardiographic parameters significantly improved. In diabetic patients a significant reduction in use of oral antidiabetic drugs and insulin was observed after 30 months of sac/val treatment. In the whole population, multivariate analysis shows that the evolution of cardiac index (CI) was significantly associated to simultaneous changes in HOMA, IGF-1 and visit; per each visit and for 1 ng/ml increase in IGF-1 there was an increase in CI of 64.77 ml/min/m^2^ (*p* < 0.0001) and 0.98 ml/min/m^2^ (*p* = 0.003), respectively, whereas 1 point increase in HOMA was associated with a −7.33 ml/min/m2 (*p* = 0.003) reduction in CI. The present data confirm persistent metabolic improvement in patients with HFrEF after treatment with sac/val and highlights its potential therapeutical role in patients with metabolic comorbidities.

## Introduction

Heart failure (HF) and type 2 diabetes mellitus (T2DM) frequently coexist, with a prevalence of T2DM as high as 35%–40% in patients with HF, independent of the degree of impairment in ejection fraction (McMurray et al., 2014a). Recent data from the Center for Medicare Services demonstrate that 55% of Medicare patients affected by HF have five or more chronic comorbidities (Tisminetzky et al., 2018). In addition, data from the European Society of Cardiology (ESC) Heart Failure Pilot Survey indicate that the majority of chronic HF patients had at least 1 comorbidity with renal disease, anemia, and T2DM being the most common ones and T2DM is the third most important comorbidity and an independent risk factor for hospital admission and death, expecially in elderly (Kristensen et al., 2016; Rosano et al., 2017; Sciacqua et al., 2021). On the other hand, according to Framingham Heart Study, diabetic patients have a 2.4-fold increased risk of HF in males and 5-fold increased risk in women regardless of other comorbidities (Artham et al., 2009; Seferovic et al., 2018). In addition, the degree of risk has further been related to the level of glycaemic control in patients with HF, and more severe hyperglycaemia has been associated with worsening of cardiac structure and function. In particular, as UKPDS has reported that for each 1% decrease in HbA1c there is a 16% decrease in risk of HF (Stratton et al., 2000). Finally, in people with T2DM, HF is a more common initial presentation of CVD than myocardial infarction (Kenchaiah and Vasan. 2015).

TD2M may cause myocardial damage indirectly through the promotion of coronary atherosclerosis, but also directly through hyperglycemia, insulin resistance, mitochondrial dysfunction, and oxidative stress involving chronic inflammation that underlies diabetic cardiomyopathy (DCM) (Jia et al., 2018; Kaur et al., 2021). In the early stages of DCM, cardiac function is preserved; however, a reduction in global longitudinal strain (GLS) can be detected. (Marwick et al., 2018; Sciacqua et al., 2019; Sciacqua et al., 2011).

In the PARADIGM-HF trial, it has been shown that compared with the angiotensin-converting enzyme inhibitor (ACEi) enalapril, sacubitril/valsartan (sac/val), an angiotensin receptor-neprilysin inhibitor (ARNI), improved morbidity and mortality in patients with HF and reduced ejection fraction (HFrEF) after a median follow-up of 27 months (McMurray et al., 2014b).

In a post hoc analysis of the PARADIGM-HF trial, it has been shown that patients with T2DM treated with sac/val exhibited an improved glycemic control as compared to those treated with enalapril at 12 months of follow up (Seferovic et al., 2017). A post-hoc analysis of the PARAGON-HF study showed that patients with HF and preserved ejection fraction (HFpEF) treated with sac/val exhibited a significantly reduction in triglyceride and an increase in HDL-cholesterol levels as compared with those treated with valsartan after a follow-up of 16 weeks (Selvaraj et al., 2021).

Natriuretic peptides (NPs), which are increased by neprilysin inhibition, might have a crucial role in insulin sensitivity and metabolism. Neprilysin is known to promote lipid mobilization from adipose tissue, increased postprandial lipid oxidation, adiponectin release, and enhanced muscular oxidative capacity (Engeli et al., 2012). According with this, blood glucose concentrations have been shown to decrease after infusion of B-type natriuretic peptide (BNP) (Heinisch et al., 2012).

In patients with chronic HF, there is an important desensitization of NPs receptors, with a significant deficiency of the active forms of NPs, leading to reduced natriuresis and diuresis, vasoconstriction, hyperactivation of the renin angiotensin aldosterone system (RAAS), and subsequent activation of the sympathetic nervous system (SNS), enhanced release of angiotensin II and aldosterone with further worsening of renal function, hemodynamic and metabolic conditions (Singh et al., 2017).

In this context, the dual inhibition of RAAS and neprilysin by sac/val effectively counteracts the neurohormonal mechanisms active in patients with HFrEF resulting in improving metabolic and hemodynamic state. A secondary analysis of the PARADIGM-HF trial has shown a beneficial effects of sac/val on renal function with patients treated with sac/val exhibiting lower decline in renal function than those treated with enalapril, an improvement that was more pronounced in diabetic patients compared to non-diabetics (Packer et al., 2018). Accordingly, a 24-month follow-up real-life study has demonstrated the efficacy and durability of sac/val treatment in patients with HFrEF not only in terms of clinical, haemodynamic and echocardiographic parameters but also regarding the renal function (Armentaro et al., 2021). Data on the improvement of metabolic parameters with sac/val treatment, in particular in diabetic patients, derive mainly from post-hoc analysis of the large clinical trials. However, whether these metabolic effects are also observed in real-life patients is still unsettled.

To this aim, we evaluate the long term effect of sac/val treatment on renal function, glycometabolic state and insulin sensitivity parameters according to diabetic status in a series of repeated observations in 90 patients with HFrEF.

## Materials and Methods

Among 150 outpatients eligible for this study, we enrolled and performed a longitudinal, observational, one-center study on 90 consecutive HFrEF Caucasian outpatients referred to the Geriatrics Department of the University Hospital of Catanzaro, 74 men and 16 females with an average age of 68 ± 10 years. The enrollment period began in March 2018 and ended in June 2018; and the follow-up period ended in December 2020. Subjects with NYHA II or III class were included by appropriate clinical tests. Other inclusion criteria were Age >18 years, LVEF <35%, treatment with stable doses of ACE-I or ARB for at least 4 weeks, according to the International Guidelines recommendations (Ponikowski et al., 2016).

According to American Diabetes Association (ADA) guidelines, diabetes mellitus can be defined as fasting plasma glucose ≥126 mg/dl, oral glucose tolerance test (OGTT) 2 h plasma glucose ≥200 mg/dl, and HbA1C ≥ 6.5%, or if antidiabetic medications were used (American Diabetes Association, 2019). No patient had clinical history of severe renal [estimated glomerular filtration rate (eGFR) <30 ml/min/1.73 m^2^] or hepatic impairement (Child-Pugh Class C), angioedema, side effects to ACE-I or ARB. None of patient was pregnant or breastfeeding, none of them had potassium levels >5.4 mmol/L or systolic blood pressure (SBP) <100 mmHg. All patients underwent an accurate medical history and a complete objective examination with the determination of the main anthropometric [weight, height, and body mass index (BMI)] and hemodynamic parameters, assessment of the NYHA functional class, quality of life with the Minnesota Living with Heart Failure Questionnaire (MLHFQ), a 12-lead electrocardiogram (ECG) using a Philips PageWriter T10 electrocardiograph and Echocardiographic recordings using a Vivid E-95 (GE Medical Systems, Milwaukee, United States) ultrasound using a 2.5 MHz transducer. Ethics Committee approved the protocol (code protocol number 2012.63) and informed consent was obtained from all participants. All investigations were carried out to accordance with the principles of the Helsinki Declaration.

Patients elegible for sac/val, in addition to their therapy after suspension of ACE-I (at least 36 h before) or ARB, received initial dosage of 24/26 mg or 49/51 mg in double administration; increasing dosage after 2–4 weeks to maximum dosage of 97/103 mg bid, according to patient tolerance. Clinical evaluation, laboratory tests, ECG, and Echocardiogram-color-Doppler were evaluated at baseline (T0) and every 6 months up to 30 months (T6, T12, T18, T24, and T30) to extimate the possible benefits and the possible occurrence of any adverse events. Furthermore, seven patients were lost to follow-up: in particular six patients died during the study, among these only three for CV causes and one patient experienced symptomatic hypotension which required withdrawal of treatment with sac-val. In addition to sac/val, the following CV drug classes were also considered: ACE-I and ARBs at baseline; while beta-blockers (BBs), mineralocorticoid receptor antagonists (MRAs), loop diuretics, statins, antiplatelet agents, and oral anticoagulants (OACs) at baseline and during follow-up; furthermore the reduction of the diuretic drugs, insulin therapy and other oral antidiabetic drugs (OADs) during the follow-up was analyzed.

### Blood Pressure

Blood pressure (BP) measurements made on the supine patient’s non-dominant arm after 5 mins of rest. Three measurements minimum were obtained in three different visits in 2 weeks one from another. The values of the SBP and diastolic BP (DBP) were recorded, respectively, in the first (phase I) and last (phase V) tone of Korotkoff. Baseline BP values represent the average of three measurements obtained at 3 min intervals.

### Echocardiograms

All patients were examined at rest and in the left lateral decubitus position. The measurements were obtained according to the international guidelines (Lang et al., 2015). Echocardiographic examinations were performed with a monoplane ultrasound probe 2.5 MHz of Vivid E-95 (GE Medical Systems, Milwaukee, United States) by a single trained operator, who was blinded to treatment protocol. Exclusively tests of excellent technical quality were used in the study, the values represented the mean of at least three and five measurements for patients with sinus rhythm and arrhythmias, respectively.

The left ventricular ejection fraction (LVEF) was calculated by the Simpson biplane method according to the following formula: LVEF = [left ventricular end-diastolic volume (LVEDV)-LV end-systolic volume (LVESV)/LVEDV*100] as mean of two measures in 4 and 2 apical chambers Both volumes were subsequently indexed for body surface area (BSA) and expressed in ml/m^2^. Cardiac output has been calculated as a continuity equation, and the cardiac index (CI) expressed in ml/min/m^2^ by means of continuity equation and the dP/dT as parameters of global systolic left ventricular function, as suggested by the guidelines (Ladipo et al., 1980; Lang et al., 2015). Left atrial volume (LAV) was measured with the area-length method and indexed for BSA (LAVI). Diastolic dysfunction was assessed by recording pulse-wave Doppler patterns at the mitral, in order to obtain early (E) and late (A) diastolic filling velocities from the 4-chamber view. Tissue Doppler imaging was performed to evaluate septal E’ and the E/E’ ratio was also calculated (Nagueh et al., 2016).

Right ventricular systolic parameters were also estimated, assessed by calculating the tricuspid annulus plane systolic excursion (TAPSE) and the systolic pulmonary artery pressure (s-PAP) estimate. The TAPSE was assessed using the M-Mode on the tricuspidal ring and expresses the longitudinal systolic function of shortening the right ventricle, a parameter assessed on the basis of ventricular interdependence. The diameter and collapsibility of the inferior vena cava (IVC) during the inhalation-expiratory phase in subcostal projection was used to estimate of the right atrial pressure. Tricuspid regurgitant velocity (TRV) was obtained by continuous Doppler at the level of the atrioventricular plane of the tricuspid valve in projection with the four apical chambers or, in the case of eccentric jets, in parasternal short axis: therefore the s-PAP was derived through the Bernoulli equation: s-PAP = 4 (TRVpeak)2 + Right atrial pressure (RAP). The evaluation of the diameter of the outflow tract of the right ventricle (RVOT) was assessed in the long axis parasternal projection. The area of the right atrium (RAA) was evaluated in apical four chambers projection (Lang et al., 2015).

For speckle tracking analysis digital loops were captured, recording at least three consecutive beats, and analyzed off-line using a dedicated software (EchoPAC 20.0; GE Medical Systems, Milwaukee, United States) by two operators who were blinded to the clinical characteristics of the patients. The same operators derived bidimensional, Doppler and speckle tracking parameters according to the most recent recommendations. If the software was not able to assess a segment due to poor image quality after manual correction of endocardial border, the segment considered as inadequate was excluded from the analysis. Briefly, each ventricular wall was analyzed into three segments with a total of 17 segments for the whole myocardium. Longitudinal strain was calculated for each segment, considering the higher value; thus the global longitudinal strain (GLS) was obtained as the mean of all 17 segments (Badano et al., 2018).

### Laboratory Determinations

All laboratory measurements were performed after a minimum fasting period of 12 h on peripheral blood samples. Uric acid and creatinine were derived by URICASE/POD and the Jaffé reaction method. To evaluate renal function, the eGFR was calculated using the CDK-EPI equation (Levey et al., 2009). NT-pro-BNP values were measured by enzyme-linked immunosorbent assay (Elecsys proBNP assay, Roche Diagnostics). Serum sodium and potassium levels were measured by indirect potentiometry (Cobas, Roche). The levels of blood glucose, triglycerides, and cholesterol were determined by enzymatic methods (Roche, Basel, and Switzerland). Plasma insulin levels were recorded by chemiluminescence method (Immulite, Siemens Healthcare, GmbH, Erlangen, and Germany). Insulin resistance was evaluated by the Homeostasis Model Assessment (HOMA) method, considering fasting insulin and glucose concentrations, according to the following formula: fasting insulin (µU/ml) × fasting glucose (mmol/L)/22.5. In addition, glycated hemoglobin (HbA1c) was measured by high performance liquid chromatography certified by the national glycohemoglobin standardization program (NGSP) and using an automatic analyzer (Adams HA-8160 HbA1c analyzer, Menarini, Italy). Insulin like growth factor-1 (IGF-1) levels were evaluated by enzyme immunoassay chemiluminescent assay (CLIA) and, finally, high sensitivity C reactive protein levels (hs-CRP) were estimated by the immunoturbidimetric method (CardioPhase hsCRP, Milan, Italy).

## Statistical Analysis

Normally distributed continuous variables were summarized as mean and standard deviation and non-normally distributed data as median and interquartile range. Categorical variables were expressed as percentages. Comparisons between patients with and without diabetes mellitus were performed by unpaired Student’s t-test for normally distributed continuous variables, Mann-Whitney U-test for non-normally distributed continuous variables or χ^2^ test for categorical data, as appropriate. The evolution of therapies across the follow up time was investigated by Cochran’s Q test. The longitudinal changes of variables were analyzed by the linear mixed model (LMM). All variables which deviate from the normal distribution were log-transformed (nl) before to be introduced into LMM as dependent variables. Multifactorial hypotheses were addressed by multiple LMMs. In these models, data were expressed as regression coefficients, 95% confident intervals (95% CIs) and *p*-values. Data analyses were performed by a commercially available statistical softwares: SPSS version 22 for Windows (Chicago, IL, United States) and STATA statistical package (version 13, TX, United States).

## Results

The study population included 90 patients attending at the Geriatrics Department at the University Hospital of Catanzaro. At baseline, the mean age was 68 ± 10 years, 82% were males and 39% active smokers (Table 1). The most frequent comorbidities associated with study population were: chronic coronary artery disease (74.4%), hypertension (83.3%), dyslipidaemia (87.7%), and T2DM (66.6%). In addition, 32.2% had valvular heart disease, 34.3% atrial fibrillation, 35.5% chronic obstructive pulmonary disease (COPD), and 45.5% of patients renal dysfunction. Finally, 47% of patients had an electronic device [implantable cardioverter defibrillator (ICD) or cardiac resynchronisation therapy defibrillator (CRTd)]. These patients had been implanted at least 12 months prior to the start of sac/val treatment. In addition, patients who met the indication for CRTd or ICD implantation during the follow-up study were not considered for data analysis. However, none of the enrolled patients met this indication.

**TABLE 1:** Baseline characteristics of patients that completed the study.

	Whole population (N = 90)	Non diabetic (N = 30)	Diabetic (N = 60)	*p*
Demographic and clinical parameters				
Age, years	68 ± 10	69 ± 10	67 ± 10	0.556
Gender (males), %	82%	83%	81%	0.845
BMI, Kg/m^2^	**32 ± 5**	**31 ± 5**	**33 ± 5**	**0.043**
Smokers, %	39%	47%	35%	0.285
Systolic BP, mmHg	122 ± 12	121 ± 12	123 ± 13	0.378
Diastolic BP, mmHg	73 ± 8	73 ± 7	73 ± 8	0.741
Heart rate, beats/min	76 ± 11	74 ± 10	77 ± 11	0.268
Respiratory rate, breath/min	18 ± 3	18 ± 3	17 ± 3	0.477
MLHFQ, total score	90 ± 4	89 ± 4	90 ± 3	0.385
Biochemical parameters				
Na, mmol/l	140.4 ± 2.2	140.4 ± 2	140.5 ± 2.4	0.755
K, mmol/l	4.4 ± 0.3	4.4 ± 0.4	4.4 ± 0.3	0.272
Creatinine, mg/dl	1.1 (0.9–1.2)	1.1 (1–1.2)	1.1 (0.9–1.3)	0.942
e-GFR, ml/min/1.73m^2^	67.3 ± 19.0	67.5 ± 17.2	67.2 ± 20	0.832
NT- proBNP, pg/ml	1904 (900–3,461)	1,608 (800–3,378)	2016 (1,050–3,488)	0.336
Fasting glucose, mg/dl	**112 (98–145)**	**101 (96–118)**	**120 (102–159)**	**0.004**
Fasting Insulin, µU/ml	27 (21–40)	25 (20–32)	29 (21.5–44.5)	0.155
IGF-1, ng/ml	82 (75.5–93.5)	82 (78–100)	82 (75–89.5)	0.662
HbA1c, %	**6.8 (5.8–7.7)**	**5.9 (5.7–6.6)**	**7.3 (6–8.2)**	**<0.001**
HOMA	**7.6 (5.6–12)**	**6.5 (5.3–9.1)**	**8.8 (5.9–16.7)**	**0.027**
Uric acid, mg/dl	6.6 ± 0.9	6.8 ± 1	6.6 ± 0.8	0.894
hs-CRP, mg/l	7.7 (7.2–7.8)	7.7 (7.1–7.8)	7.7 (7.3–7.8)	0.747
LDL cholesterol, mg/dl	78 (56.6–94.6)	80.5 (61.4–94.4)	75 (55.9–94.6)	0.697
HDL cholesterol, mg/dl	81.3 ± 33.5	44.4 ± 8.6	42.2 ± 10.6	0.231
Triglycerides, mg/dl	**130.5 (110–189)**	**114.5 (93–150)**	**132.5 (112–190)**	**0.041**
Echocardiographic parameters				
LVEDV/BSA, ml/m^2^	**87.8 (83.1–95)**	**92 (87–98.3)**	**85.5 (82–93.4)**	**0.007**
LVESV/BSA, ml/m^2^	**60.4 (56–65.2)**	**63 (60–68)**	**58 (56–64)**	**0.003**
LVEF, %	31.8 (30.8–32.9)	31.3 (30.5–32.7)	31.9 (31.2–33.2)	0.084
Cardiac index, ml/min/m^2^	1,667 (1,534–1887)	1,656 (1,569–1807)	1,667 (1,529–1903)	0.467
E/e’	17 (16–18)	17.5 (16–18)	17 (16–18)	0.084
GLS, %	−7.8 (from −8.8 to −7)	−7.9 (from −8.9 to −7)	−7.7 (from −8.75to −7)	0.587
TAPSE, mm	16 (15.5–17)	16 (15–17)	16 (16–17)	0.589
s-PAP, mmHg	44.5 (41–48)	44.5 (42–48)	44.5 (40–48)	0.612
IVC, mm	19.6 (19.4–21)	19.7 (19.5–21)	19.6 (19.4–21)	0.615

BMI, body mass index; BP, blood pressure; MLHFQ, minnesota living with heart failure questionnaire; Na, Sodium; K, potassium; e-GFR, estimated glomerular filtration rate; NT-proBNP, N-terminal pro-brain natriuretic peptide; IGF-1, insulin-like growth factor-1; HbA1c, glycated haemoglobin; HOMA, homeostatic model assessment; hs-CRP, highly sensitive c-reactive protein; HDL, high density lipoprotein; LDL, low density lipoprotein; LVEDV/BSA, left ventricular end-diastolic volume index/body surface area; LVESV/BSA, left ventricular end-systolic volume index/body surface area; LVEF, left ventricular ejection fraction; GLS, global longitudinal strain; TAPSE, Tricuspid annular plane systolic excursion; s-PAP, systolic pulmonary arterial pressure; IVC, inferior vena cava.

Variables that differed significantly between the two groups in the study population at baseline are shown in bold.

Baseline clinical, biochemical and echocardiographic characteristics of the study population are reported in Table 1.

The study group was stratified according to diabetes mellitus. Sixty patients had history of T2DM (66.6%), while the remaining 30 (33.4%) did not have T2DM. These two groups of patients had similar baseline characteristics except for BMI, fasting glucose, HbA1c, HOMA, and triglycerides values that were significantly higher in diabetic than in non-diabetic patients (Table 1). In addition, patients with T2DM showed lower values of IGF-1, LVEDV, and LVESV indexed for BSA (Table 1).

At baseline, 62.2% of patients started the lowest dose of sac/val (100 mg/die), and 37.8% 200 mg/die. At 30-months of follow-up, 19% of patients were taking the lowest dose of sac/Val (100 mg/die), 55% of the patients the intermediate dose (200 mg/die), and 26% the highest dose (400 mg/die) (Table 2). Regarding NYHA functional class, at baseline 58% of patients had NYHA class III and 42% NYHA class II. At 30 months follow-up, 7.8% of patients reverted to NHYA class I, 82.2% were in NHYA class II, and 10% remained in NYHA class III. Regarding adverse events, there were only four episodes of symptomatic hypotension but only one required drug discontinuation.

**TABLE 2:** Evolution of therapies across time.

	Time (months)						
	0 (%)	6 (%)	12 (%)	18 (%)	24 (%)	30 (%)	p*
MRAs	**50**	**41**	**37**	**34**	**31**	**30**	**<0.001**
Statins	78	78	78	79	78	79	1.000
Beta-blockers	99	99	99	99	99	99	1.000
OACs	33	33	33	32	32	32	1.000
Antiplatelet therapy	57	57	57	58	58	56	1.000
Diuretics	**100**	**90**	**86**	**82**	**79**	**78**	**<0.001**
OADs**	**100**	**92**	**83**	**79**	**72**	**65**	**<0.001**
Insulin therapy**	**30**	**27**	**24**	**21**	**18**	**13**	**<0.001**
Sac/val Dose mg							
100	**62**	**20**	**20**	**19**	**19**	**19**	**<0.001**
200	**38**	**58**	**58**	**58**	**58**	**55**	
400		**22**	**22**	**23**	**23**	**26**	

p*derived by Test di Cochran’ Q on listwise; derived by Friedman test for entresto doses. MRAs, mineralocorticoid receptor antagonists; OACs, oral anticoagulants; OADs, oral antidiabetic drugs; Sac/val, Sacubitril-Valsartan. **only on diabetic patients.

The percentage changes in drug classes taken by patients at baseline and during follow-up are shown in bold.

### Evolution of Pharmacological Treatment Over Time

The main drugs taken by the patients and their evolution during follow-up are described in Table 2. At baseline 50% of patients were taking mineral receptor antagonists (MRA), whose intake decreased by 20% at 30 months of follow-up (*p* < 0.001); and 100% of patients were taking loop diuretics, which showed a statistically significant reduction of 22% of intake at the follow-up (*p* < 0.001).

At baseline, all patients with T2DM were treated with oral antidiabetic drugs (OADs), whereas at 30 months follow up only 65% were still taking OADs. In addition, at baseline 30% of diabetic patients were treated with insulin whereas at 30 months follow up only 13% continued the insulin treatment. At baseline, 18 (30%) diabetic patients were treated with OADs and insulin concomitantly, while at 30 months only 8 (13%). None of the patients with T2DM were treated with glucagon-like peptide 1 receptor agonist (GLP-1 RA) and only three (5%) patients were treated with sodium glucose cotransporter 2 inhibitors (SGLT2i).

### Evolution of Study Biomarkers Over Time

During the follow-up period there was a significant improvement in clinical, echocardiographic and in particular laboratory parameters (Table 3). A significant decrease in BMI from 32 ± 5 to 29 ± 4 kg/m^2^ was observed, *p* < 0.001. There was also a marked decrease in heart rate (HR) from 76 ± 11 to 64 ± 6 beats/min, *p* < 0.001, and in respiratory rate (RR) from 18 ± 3 to 13 ± 1 breath/min, *p* < 0.001. SBP also decreased from 122 ± 12 to 113 ± 7 mmHg, *p* < 0.001 as well as DBP from 73 ± 8 to 66 ± 6 mmHg, *p* < 0.001. In addition, the introduction of sac/val therapy led to an improvement in several echocardiographic parameters, with a reduction in left ventricular volumes: LVEDV/BSA from 89.4 ± 8.2 to 82.3 ± 10.3 ml/m^2^, *p* < 0.001; LVESV/BSA from 60.9 ± 5.9 to 50.0 ± 6. 5 ml/m^2^, *p* < 0.001; resulting in an increase in LVEF 31.8% ± 1.4% vs. 39.3% ± 1.6%, *p* < 0.001; in CI from 1698.1 ± 201.4 ml/min/m^2^ to 2158.8 ± 211.1 ml/min/m^2^, *p* < 0.001; and in left ventricular myocardial deformation indices: GLS from −7.8% ± 1.2% to −13.9% ± 1.7%, *p* < 0.001. In addition, there was also an improvement in right heart parameters, in particular an increase in TAPSE from 16.2 ± 1.3 mm to 19.3 ± 2.3 mm, *p* < 0.001 and a decrease in s-PAP 45.4 ± 7.3 mmHg vs. 33.6 ± 4.9 mmHg, *p* < 0.001. All these findings indicate a reduction in systemic and pulmonary congestion as confirmed by the decrease in NT-proBNP median values 1,904 (900–3,461) pg/ml vs. 628.5 (389–1,245) pg/ml, *p* < 0.001; IVC diameter 19.6 (19.4–21) mm vs. 18 (17–19) mm, *p* < 0.001. Finally, there was also a reduction in left ventricular filling pressures E/e' from 17 (16–18) to 14 (13–15), *p* < 0.001. The amelioration of haemodynamic conditions may justify, through increase in systemic flow and renal perfusion, the improvement in eGFR from 67.3 ± 19 ml/min/1.73 m^2^ to 86.4 ± 13.2 ml/min/1.73 m^2^, *p* < 0.001 with a rate of increase of 3.89 ml/min/1.73 m^2^ (95% CI: 3.12–4.66) every 6 months.

**TABLE 3:** Linear mixed models of study variables over time.

	Time (months)						
	0	6	12	18	24	30	P
BMI, Kg/m^2^	32 ± 5	31 ± 5	30 ± 5	30 ± 5	29 ± 5	29 ± 4	<0.001
Systolic BP, mmHg	122 ± 12	119 ± 12	118 ± 10	116 ± 8	115 ± 8	113 ± 7	<0.001
Diastolic BP, mmHg	73 ± 8	70 ± 7	69 ± 7	67 ± 7	66 ± 6	66 ± 6	<0.001
Heart rate, beats/min	76 ± 11	72 ± 8	69 ± 8	66 ± 7	65 ± 7	64 ± 6	<0.001
Respiratory rate, breath/min	17 ± 3	16 ± 2	15 ± 2	13 ± 2	13 ± 2	13 ± 1	<0.001
MLHFQ, total score	89.7 ± 3.6	84.1 ± 4.9	80.5 ± 4.4	77.4 ± 4.5	75.3 ± 3.7	73.1 ± 3.9	<0.001
e-GFR, ml/min/1.73 m^2^	67.3 ± 19	72.8 ± 17.9	81.4 ± 17.5	83.8 ± 15.8	85.5 ± 16	86.4 ± 13.2	<0.001
NT- proBNP, pg/ml	1904 (900–3,461)	1,281 (678–2,873)	979 (432–1845)	770 (411–1,670)	712 (411–1,476)	628.5 (389–1,245)	<0.001
Glycemia, mg/dl	112 (98–145)	102 (92–136)	95 (89–101)	91 (88–100)	90 (85–96)	88.5 (81–93)	<0.001
Insulinemia, µU/ml	27 (21–40)	22 (18–35)	19.5 (15–25)	19 (15–23)	18 (15–20)	17 (15–19)	<0.001
IGF-1, ng/ml	82 (76–92)	88 (81–106)	99.5 (90–116)	103 (96–125)	109 (100–129)	116 (106–134)	<0.001
HbA1c, %	6.8 (5.8–7.7)	5.9 (5.4–7.5)	5.6 (5–6.2)	5.6 (5–6.2)	5.6 (5–6.2)	5.4 (5–6)	<0.001
HOMA, (mmol/L*[μU/ml]/22.5)	7.55 (5.63–11.93)	5.99 (4.44–9.41)	4.71 (3.64–6.42)	4.44 (3.64–5.87)	4.13 (3.44–5.21)	3.75 (3.08–4.19)	<0.001
Uric acid, mg/dl	6.63 ± 0.89	5.9 ± 1.08	5.71 ± 1.12	5.62 ± 0.99	5.49 ± 0.82	5.41 ± 0.75	<0.001
hs-CRP, mg/l	7.65 (7.2–7.8)	6.8 (6.4–7)	6.175 (5.74–6.31)	5.61 (5.27–5.74)	5.02 (4.61–5.15)	4.33 (3.78–4.61)	<0.001
LDL cholesterol, mg/dl	81 ± 33	77 ± 30	72 ± 28	71 ± 29	69 ± 20	67 ± 19	<0.001
HDL cholesterol, mg/dl	43 ± 10	44 ± 9	45 ± 12	46 ± 11	47 ± 10	46 ± 9	<0.001
Triglycerides, mg/dl	130.5 (110–189)	128.5 (100–167)	110 (88–125)	110 (88–121)	110 (87–115)	99.5 (84–106)	<0.001
LVEDV/BSA, ml/m^2^	89.48 ± 8.24	87.97 ± 7.67	85.01 ± 10.16	83.4 ± 10.33	82.9 ± 10.33	82.34 ± 10.35	<0.001
LVESV/BSA, ml/m^2^	60.96 ± 5.9	57.75 ± 5.32	54.6 ± 6.53	51.83 ± 6.62	50.88 ± 6.61	50.01 ± 6.56	<0.001
LVEF, %	31.88 ± 1.47	34.37 ± 1.53	35.76 ± 1.59	37.87 ± 1.65	38.66 ± 1.67	39.3 ± 1.6	<0.001
Cardiac index, ml/min/m^2^	1,698.19 ± 201.49	1879.83 ± 205.74	1972.97 ± 200.05	2029.97 ± 209.63	2,103.27 ± 209.63	2,158.89 ± 211.15	<0.001
E/e’	17 (16–18)	16 (14–17)	15 (13–16)	14 (13–15)	14 (13–15)	14 (13–15)	<0.001
GLS, %	−7.81 ± 1.26	−9.04 ± 1.6	−10.85 ± 1.54	−11.64 ± 2.1	−12.88 ± 2.06	−13.92 ± 1.73	<0.001
TAPSE, mm	16.2 ± 1.3	17.1 ± 1.8	17.8 ± 1.8	18.2 ± 2.1	19 ± 2.5	19.3 ± 2.3	<0.001
s-PAP, mmHg	45.4 ± 7.3	42.4 ± 7.3	40.4 ± 7.3	37.5 ± 6.3	34.6 ± 5.6	33.6 ± 4.9	<0.001
IVC, mm	19.6 (19.4–21)	19 (17–21)	19 (18–20)	18 (18–20)	18 (18–20)	18 (17–19)	<0.001

BMI, body mass index; BP, blood pressure; MLHFQ, minnesota living with heart failure questionnaire; Na, Sodium; K, potassium; e-GFR, estimated glomerular filtration rate; NT-proBNP, N-terminal pro-brain natriuretic peptide; IGF-1, insulin-like growth factor-1; HbA1c, glycated haemoglobin; HOMA, homeostatic model assessment; hs-CRP, highly sensitive c-reactive protein; LDL, low density lipoprotein; HDL, high density lipoprotein; LVEDV/BSA, left ventricular end-diastolic volume index/body surface area; LVESV/BSA, left ventricular end-systolic volume index/body surface area; LVEF, left ventricular ejection fraction; GLS, global longitudinal strain; TAPSE, Tricuspid annular plane systolic excursion; s-PAP, systolic pulmonary arterial pressure; IVC, inferior vena cava.

Notably, during the entire follow-up period, we observed marked changes of several metabolic parameters with improvement of glycemic control and insulin resistance. Fasting glucose decreased from 112 (98–145) to 88.5 (81–93) mg/dl, *p* < 0.001, fasting insulin from 27 (21–40) to 17 (15–19) μU/ml, *p* < 0.001, and HOMA index from 7.5 (5.6–11.9) to 3.7 (3.0–4.1), *p* < 0.001. There was also a reduction in HbA1c values from 6.8 (5.8–7.7)% to 5.4 (5–6)%, *p* < 0.001, and an increase in IGF-1 values 82 (76–92) vs. 116 (106–134) ng/ml, *p* < 0.001, with a rate increase of 6.78 ng/ml (95% CI: 5.84–7.72) per semester indicating an improvement in insulin sensitivity. Also lipid parameters significantly improved, in particular HDL-cholesterol increased, whereas LDL-cholesterol and triglycerides significantly decreased (Table 3). hs-CRP decreased from 7.6 (7.2–7.8) to 4.3 (3.7–4.6) mg/L, *p* < 0.001, with a significant reductions of −0.68 mg/L per semester (95% CI: from −0.72 to −0.64 together with a reduction in uric acid values from 6.6 ± 0.8 mg/dl to 5.4 ± 0.7 mg/dl, *p* < 0.001.

**FIGURE 1:**
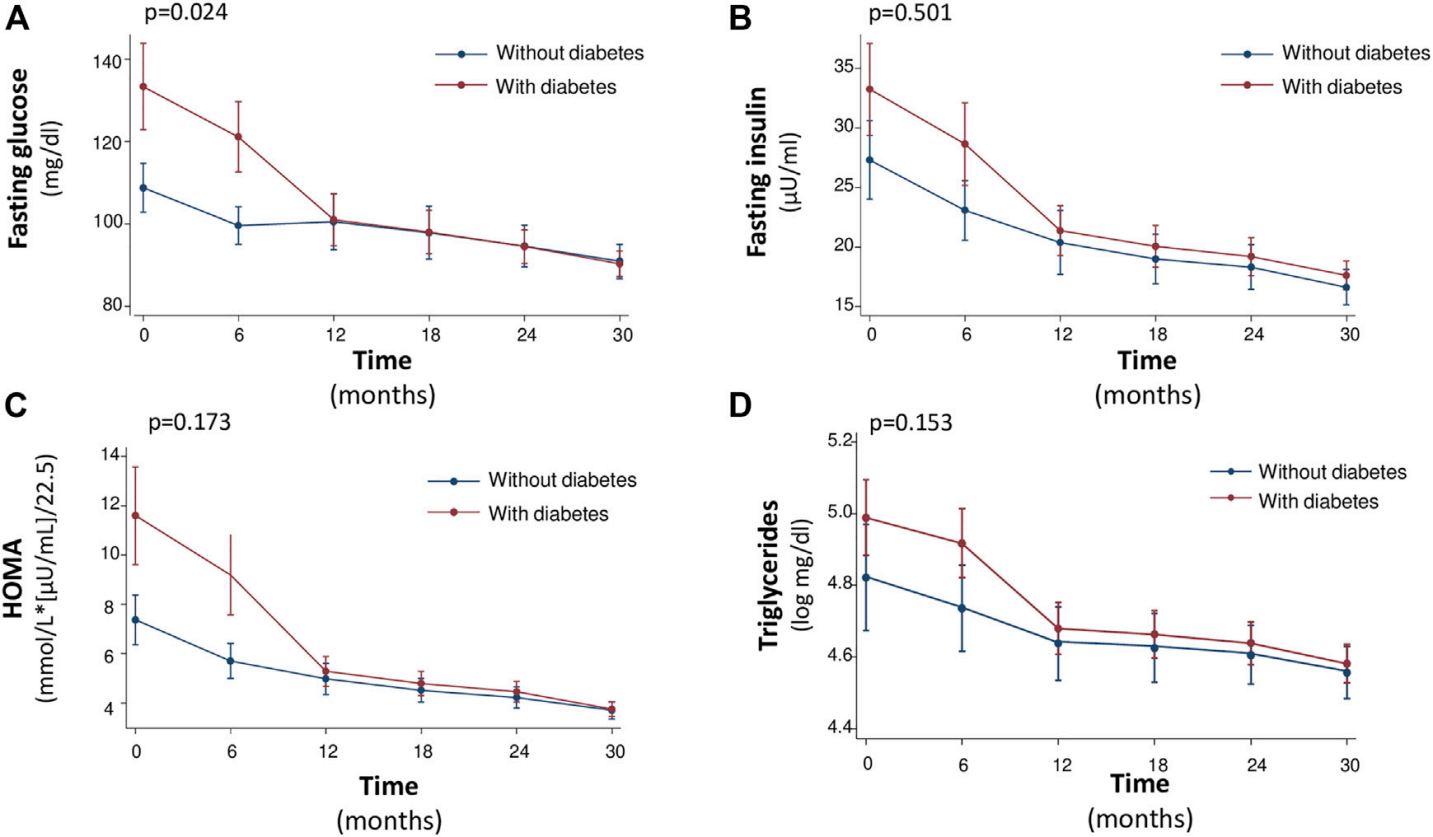
Median values of Fasting glucose (mg/dl) **(A)**, Fasting Insulin (μU/ml) **(B)**, HOMA [mmol/L*(μU/ml)/22.5] **(C)** and Triglycerides (log mg/dl) **(D)** at baseline and every 6 months, during the 30-month follow-up.

**FIGURE 2:**
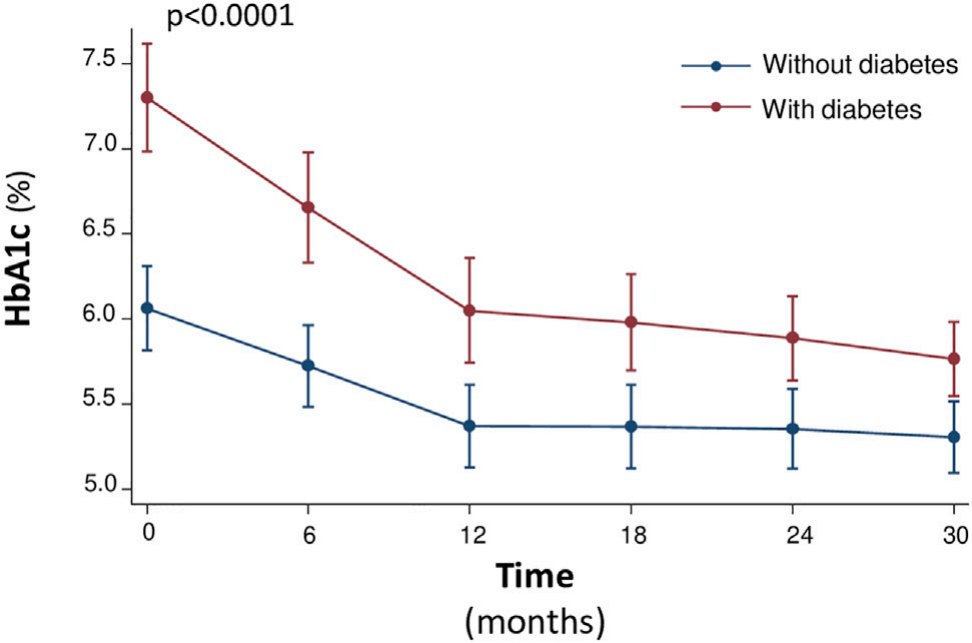
Median values of the HbA1c (%) at baseline and every 6 months, during the 30-month follow-up.

Notably, the rate of improvement of glycometabolic parameters in particular HbA1c, triglycerides, fasting glucose, insulin and HOMA was significantly steeper in patients with T2DM that in non-diabetic patients (Figures 1, 2). In particular, fasting glucose, insulin and HOMA in patients with T2DM achieved almost the same values of non-diabetics from 12th month onwards. In addition the reduction of HbA1c and triglycerides was of higher magnitude in diabetic than in non-diabetic patients from baseline to 12th month, with a plateau observed from 12th month onwards.

### Independent correlates of repeated measurements of left ventricular end-diastolic volume/BSA, LVESV/BSA, GLS, and Cardiac index

To assess the independent correlates of LVEDV/BSA, LVESV/BSA, GLS, and CI over time, a multiple linear mixed model was applied including a number of covariates, specifically the different metabolic parameters and the impact of the visit at every follow-up. In multivariate LMMs, the longitudinal changes of LVEDV/BSA were significantly associated to concomitant changes of IGF-1 and visit. In fact, for each visit and for each 1 ng/ml increase in IGF1, a −1.3 ml/m^2^ (*p* < 0.001) and −0.03 ml/m^2^ (*p* = 0.03) reduction in LVEDV/BSA over time was observed, respectively (Table 4). Furthermore, the changes of LVESV/BSA were significantly related to simultaneous changes of eGFR, uric acid, HbA1c, and visit; in fact, for each visit and for each 1 ml/min/1.73 m^2^ increase in eGFR, a −1.87 ml/m^2^ (*p* < 0.001) and −0.03 ml/m^2^ (*p* = 0.05) reduction in LVESV/BSA was observed, respectively, whereas a 1 mg/dl increase in uric acid and a 1% increase in HbA1c were associated with a 0.49 ml/m^2^ (*p* = 0.045) and 0.32 ml/m^2^ (*p* = 0.042) increase in LVESV/BSA, respectively (Table 5). The eGFR was also a correlate of GLS over time together with BMI and visit; in fact, for each visit and for each 1 ml/min/1.73 m^2^ increase in eGFR, a −1.08% (*p* = 0.001) and −0.01% (*p* = 0.026) reduction in GLS was observed, respectively, whereas a 1 kg/m^2^ increase in BMI was associated with a 0.08% increase in GLS (Table 6). Finally, the evolution of CI was significantly associated to simultaneous changes in BMI, HOMA, IGF-1, and visit; per each visit and for 1 ng/ml increase in IGF-1 there was an increase in CI of 64.77 ml/min/m^2^ (*p* < 0.0001) and 0.98 ml/min/m^2^ (*p* = 0.003), respectively, whereas a 1 kg/m^2^ increase in BMI and 1 point increase in HOMA were associated with a −7.27 ml/min/m^2^ (*p* = 0.029) and −7.33 ml/min/m^2^ (*p* = 0.003) reduction in CI, respectively (Table 7).

**TABLE 4:** Multivariate linear mixed models of LVEDV/BSA over time.

	Regression coefficient (95%CI)	*p*-value
Visit	−1.30 (from −1.54 to −1.06)	<0.001
BMI, Kg/m^2^	−0.03 (from −0.31 to 0.25)	0.854
HOMA, (mmol/L*[μU/ml]/22.5)	−0.07 (from −0.16 to 0.01)	0.086
e-GFR, ml/min/1.73 m^2^	−0.03 (from −0.08 to 0.02)	0.260
Uric acid, mg/dl	0.49 (from −0.14 to 1.11)	0.128
IGF-1, ng/ml	−**0.03 (from** −**0.06 to 0.003)**	**0.030**
HbA1c, %	0.16 (from −0.24 to 0.56)	0.425

BMI, body mass index; HOMA, homeostatic model assessment; e-GFR, estimated glomerular filtration rate; IGF-1, insulin-like growth factor-1; HbA1c, glycated haemoglobin.

The dependent variables that significantly correlate with the dependent variable of each Multivariate linear mixed models are shown in bold.

**TABLE 5:** Multivariate linear mixed models of LVESV/BSA over time.

	Regression coefficient (95%CI)	*p*-value
Visit	−1.87 (from −2.04 to −1.70)	<0.001
BMI, Kg/m^2^	0.12 (from −0.07 to 0.31)	0.215
HOMA, (mmol/L*[μU/ml]/22.5)	−0.04 (from −0.10 to 0.03)	0.282
e-GFR, ml/min/1.73 m^2^	−**0.03 (from** −**0.07 to 0.0003)**	**0.050**
Uric acid, mg/dl	**0.49 (from 0.01 to 0.96)**	**0.045**
IGF-1, ng/ml	−0.02 (from −0.04 to 0.001)	0.063
HbA1c, %	**0.32 (from 0.01 to 0.63)**	**0.042**

BMI, body mass index; HOMA, homeostatic model assessment; e-GFR, estimated glomerular filtration rate; IGF-1, insulin-like growth factor-1; HbA1c, glycated haemoglobin.

The dependent variables that significantly correlate with the dependent variable of each Multivariate linear mixed models are shown in bold.

**TABLE 6:** Multivariate linear mixed models of GLS over time.

	Regression coefficient (95%CI)	*p*-value
Visit	−1.08 (from −1.19 to −0.96)	0.000
BMI, Kg/m^2^	0.08 (from 0.04 to 0.12)	0.000
HOMA, (mmol/L*[μU/ml]/22.5)	0.002 (from −0.03 to 0.03)	0.878
e-GFR, ml/min/1.73m^2^	−**0.01 (from** −**0.02 to** −**0.00)**	**0.026**
Uric acid, mg/dl	0.13 (from −0.05 to 0.31)	0.149
IGF-1, ng/ml	−0.003 (from −0.009 to 0.004)	0.412
HbA1c, %	0.10 (from −0.08 to 0.27)	0.283

BMI, body mass index; HOMA, homeostatic model assessment; e-GFR, estimated glomerular filtration rate; IGF-1, insulin-like growth factor-1; HbA1c, glycated haemoglobin.

The dependent variables that significantly correlate with the dependent variable of each Multivariate linear mixed models are shown in bold.

**TABLE 7:** Multivariate linear mixed models of CI over time.

	Regression coefficient (95%CI)	*p*-value
Visit	**64.77 (from 57.67 to 71.87)**	**0.000**
BMI, Kg/m^2^	−**7.27 (from** −**13.78 to** −**0.76)**	**0.029**
HOMA, (mmol/L*[μU/ml]/22.5)	−**6.33 (from** −**10.12 to** −**2.53)**	**0.001**
e-GFR, ml/min/1.73 m^2^	0.63 (from −0.25 to 1.51)	0.161
Uric acid, mg/dl	−12.75 (from −28.04 to 2.53)	0.102
IGF-1, ng/ml	**0.98 (from 0.34 to 1.62)**	**0.003**
HbA1c, %	−3.34 (from −19.98 to 13.29)	0.694

BMI, body mass index; HOMA, homeostatic model assessment; e-GFR, estimated glomerular filtration rate; IGF-1, insulin-like growth factor-1; HbA1c, glycated haemoglobin.

The dependent variables that significantly correlate with the dependent variable of each Multivariate linear mixed models are shown in bold.

### Evolution of Variables Over Time Between Diabetics and Nondiabetics

Moreover, during the study there was a different evolution over time of the natural logarithm (nl) of fasting glucose (*p* = 0.024), fasting insulin (0.501), HOMA (*p* = 0.173), and triglycerides (*p* = 0. 153) in diabetic versus nondiabetic patients, according with this a different trend was indeed observed until or at 12 months after initiation of sac/val therapy, after which the trend of these variables was similar between diabetics and nondiabetics, see Figure 1. On the other hand, HbA1c had a different trend between the two groups with respect to the other variables. In fact, there was an important reduction in the natural logarithm of HbA1c in both groups up to 12 months, after 12 months the variation of the variable was not superimposable between the two groups (*p* < 0.0001), see Figure 2.

## Discussion

In this longitudinal-observational, single-center study, considering 90 HFrEF patients with optimal medical therapy but still symptomatic, sac/val treatment demonstrated efficacy, safety and durability every 6 months up to 30 months of follow-up, with an important improvement in clinical and echocardiographic parameters confirming previous evidence (Armentaro et al., 2021). Notably, sac/val showed also a significant betterment in renal function, glycometabolic state and insulin sensitivity parameters and this effect was particularly noticeable in T2DM patients who represented about 67% of the entire population.

After a 30-month follow-up, 81% of the patients was taking the intermediate/high dose of sac/val and an important improvement in both NYHA class and MLHFQ score was observed. These findings were related to the hemodynamic state improvement as demonstrated by the positive changes of the morphological and functional right and left myocardial parameters and by the reduction of pulmonary and systemic venous congestion as demonstrated by decreased NT-proBNP levels and lower use of loop diuretics and MRAs.

The favorable effect of sac/val on renal function has been well demonstrated in the PARADIGM-HF study, in fact patients treated with sac/val showed a slower decline in eGFR compared to enalapril and the benefit was higher in diabetics than in non-diabetics (McMurray et al., 2014a; Packer et al., 2018) and confirmed in a recent meta-analysis.

In the present study, improvement in renal function is already significant after 6 months, consistent with our previous work (Pelaia et al., 2022), and mean changes in e-GFR remain statistically significant until 30 months. If at least part of the eGFR increase can be ascribed to betterment in hemodynamic conditions and renal blood flow by the contemporary modulation of RAAS and NEP, that leads to reduction of intraglomerulary pressure and direct antioxidant, anti-inflammatory and antifibrotic effects respectively, however it may be due to a direct protective effect on the kidney (Judge et al., 2015) (Ruggenenti and Remuzzi, 2015). This finding is clinically relevant because renal dysfunction has an important prognostic role in HF patients (Damman et al., 2014).

The improvement of renal function and the progressive reduction in the use of loop diuretics and MRAs may, at least in part, justify an important reduction in uric acid levels, a known cardiovascular risk factor, especially when associated with other metabolic abnormalities so as insulin resistance (Cassano et al., 2020).

Notably, during the follow-up period significant changes in metabolic parameters, together with hs-CRP levels reduction, have been also observed. In particular, fasting glucose, insulin and HbA1c levels significantly reduced with lowering of HOMA and increase of IGF-1 levels, thus indicating a significant betterment of insulin sensitivity. According with this, also lipid profile significantly improved with reduction of LDL-cholesterol and triglycerides and increase of HDL-cholesterol. Taken together, this better glycemic control accounted for the reduction of OADs and insulin therapy use. Of interest, the improvement of glycometabolic parameters was more evident in diabetic patients and their values achieved almost the same value of non-diabetics from 12th months onwards, indicating a greater effective of the treatment in diabetic setting. Moreover, the longitudinal changes of LV volumes, GLS and CI were significantly associated to simultaneous variations of BMI, HOMA, and IGF-1 and this association was consistent for every visit; emphasizing the effect of the drug on glyco-metabolic parameters and how these determine an important improvement in cardiac volumes, function and global left myocardial strain indices.

Our data are in agreement with results of a post-hoc analysis from the PARADIGM-HF trial showing that treatment with sac/val for the 1st year resulted in a significant reduction in HbA1c in comparison with enalapril. This benefit persisted during the follow-up as demonstrated by a 23% and a 29% reduction in new OADs and insulin therapy use, respectively (Seferovic et al., 2017). This is particularly relevant because patients with a history of diabetes or with previously undiagnosed diabetes, had a 38% greater risk of CV death or HF hospitalization than those without diabetes, moreover an increased risk for poor outcome is also observed in pre-diabetes than non-diabetic patients (HbA1c < 6.0%) (Kristensen et al., 2016).

Thus, this additional effect of sac-val on glycemic control and insulin sensitivity parameters makes this drug not only safe and effective on symptoms control and cardiac reverse remodeling in patients with HFrEF, but also suggests a possible favorable metabolic effect in the entire population with particular regard to diabetic subjects (de Diego et al., 2018; Martens et al., 2019).

In our study, there is also a significant reduction in LDL cholesterol and triglyceride levels and a modest increase in HDL cholesterol levels. This is not surprising, in fact a post-hoc analysis of the PARAGON-HF study showed that HF patients with preserved ejection fraction (HFpEF) treated with sac/val compared with valsartan at 16 weeks from baseline had significantly lower triglycerides and higher HDL-cholesterol levels. These metabolic effects were directly related to NPs activity in fact they were no longer evident after adjustment for urinary cGMP/creatinine (a biomarker of natriuretic peptide activation). However, in addition to these beneficial effects, an increase in LDL-cholesterol, that cannot be attributed to sac/val, was observed. (Selvaraj et al., 2021). However, PARAGON-HF analysis was conducted considering a follow-up of only 16 weeks, on the contrary in the present real life study, we observed the increase in HDL cholesterol levels and a significant reduction in triglycerides and LDL cholesterol levels during a 30-month follow-up.

Even if the positive metabolic effects of sac/val treatment could be partly justified by the reduction of diuretic drugs and by the improvement in quality of life and NYHA class which could have favored an increase in physical activity, however the modulation of RAAS and NPs system by sac/val may affect directly several pathophysiological mechanisms.

In particular, it's known that sustained RAAS activation and NPs deficiency are common conditions in HF thus promoting a proinflammatory state, metabolic dysfunction and IR. Sac/val may improve the glycometabolic profile and insulin sensitivity through both inhibition of NEP and blocking angiotensin II type 1 receptors. NEP is practically ubiquitous, in fact it is abundantly expressed, as well as in the kidney, also in adipocytes, pancreatic islets, cardiomyocytes, and endothelial vascular cells; furthermore NEP recognizes about fifty substrates therefore its inhibition can have important implications. In particular, the increase of NPs levels can favour lipid mobilization and postprandial oxidation, increased adiponectin synthesis and browing of white adipose tissue, all mechanisms that promote insulin sensitivity. Moreover also other substances are affected by NEP inhibition, in particular bradykinin and glucagon like peptide-1 (GLP-1) increase and endothelin-1 decrease. This is clinically relevant because bradykinin may positively affect insulin signaling, glucose uptake, free fatty acids synthesis, and adiponectin expression in adipose tissue thus improving insulin sensitivity and glycemic control. In addition, GLP-1 may regulate insulin secretion and stimulate extrapancreatic glucoregulation; moreover it may affect appetite and food intake. Finally, lower edothelin-1 levels are associated with reduced lipolysis and improved insulin sensitivity (Giamouzis and Butler, 2017; Singh et al., 2017; Seferovic et al., 2020).

Taken together, the results of the present study might suggest that the improvement of inflammatory and metabolic conditions during sac/val treatment could represent another important mechanism by which the drug exerts its positive effects on cardiac reverse remodeling and hemodynamic balance. These additional metabolic effects of sac/val may have clinical relevance for several reasons; in particular the improvement in insulin sensitivity and HbA1c may favour a reduced use of harmful OADs, such as sulfanilureas, particularly in elderly (Giamouzis and Butler, 2017; Sciacqua et al., 2021). Moreover the progressive reduction in OADs and insulin therapy may reduce drug interactions improving compliance to pharmacological treatment.

The strengths of this study are represented by the fact that it is a real life study with patients showing important comorbidities and a polypharmacological therapy compared to RCTs. In addition, during a fairly consistent follow-up of 30 months sac/val treatment confirmed not only safety but also efficacy and durability, showing also new potential metabolic effects, that were never evaluated in real life studies before. Furthermore, considering that none of diabetic patients were treated with GLP-1 RA and only 5% of patients were treated with SGLT2i, OADs particularly relevant on metabolic and CV profile, it’s possible hypothesize that the long-term changes in the study variables can be predominantly attributed to sac/val.

The present study have also several limitations; at first it is not a randomized clinical trial and a matched control group is not available. However, we can consider each patient as a control of himself as before enrollment every patient was treated with the best possible therapy according to current guidelines, but patients were still symptomatic. Other important limitations are represented by the relatively small sample size and the lack of information regarding the physical activity level of enrolled patients. The study protocol did not contemplate a sample size calculation and for this reason our findings, particularly those which did not attain the statistical significance, should be interpreted with very caution. The imbalance of the number of patients between diabetic and non-diabetic patients represents an intrinsic limitation of our study because a 1:1 ratio between the two groups (instead of 2:1) would have been the ideal one. In addition, the reduction of systemic and pulmonary congestion was assessed clinically and echocardiographically and not by a more accurate method such as: bioelectrical impedance vector analysis (Massari et al., 2018). A final limitation is the low percentage of patients on Dapagliflozin or Empagliflozin therapy.

## Conclusion

To our knowledge, this real-life study is the only one with 30-month follow-up in which the safety, efficacy, and durability of sac-val were demonstrated throughout the follow-up period with a good tolerability profile. Furthermore, this is the first study in which not only the long-term effects of sac-val on reverse remodeling, but also on glycemic control, insulin-sensitivity, lipid parameters, and renal function have been evaluated. Considering present findings, the study results support the need to initiate sac/val treatment as early as possible and optimizing HF treatment particularly before ICD implantation, as recommended by current guidelines (McDonagh et al., 2021). An early use of sac/val in HFrEF patients with numerous comorbidities, in particular T2DM, would allow not only to improve QoL, but also to act on the pathophysiological mechanisms of the disease, promoting reverse cardiac remodeling, preserving renal function and improving insulin resistance and dyslipidemia thus allowing a positive and substantial intervention on prognosis.

## Data Availability

The raw data supporting the conclusion of this article will be made available by the authors, without undue reservation.
